# Septin‐regulated actin dynamics promote *Salmonella* invasion of host cells

**DOI:** 10.1111/cmi.12866

**Published:** 2018-07-26

**Authors:** Kirsten C. Boddy, Aggie Duan Gao, Dorothy Truong, Moshe S. Kim, Carol D. Froese, William S. Trimble, John H. Brumell

**Affiliations:** ^1^ Cell Biology Program Hospital for Sick Children Toronto Canada; ^2^ Department of Molecular Genetics University of Toronto Toronto Canada; ^3^ Institute of Medical Science University of Toronto Toronto Canada; ^4^ Department of Biochemistry University of Toronto Toronto Canada; ^5^ SickKids IBD Centre Hospital for Sick Children Toronto Canada

**Keywords:** actin, bacterial infection, formin, myosin II, *Salmonella enterica* serovar Typhimurium, septin

## Abstract

Actin nucleators and their binding partners play crucial roles during *Salmonella* invasion, but how these factors are dynamically coordinated remains unclear. Here, we show that septins, a conserved family of GTP binding proteins, play a role during the early stages of *Salmonella* invasion. We demonstrate that septins are rapidly enriched at sites of bacterial entry and contribute to the morphology of invasion ruffles. We found that SEPTIN2, SEPTIN7, and SEPTIN9 are required for efficient bacterial invasion. Septins contributed to the recruitment of ROCK2 kinase during *Salmonella* invasion, and the downstream activation of the actin nucleating protein FHOD1. In contrast, activation of the ROCK2 substrate myosin II, which is known to be required for *Salmonella enterica* serovar Typhimurium invasion, did not require septins. Collectively, our studies provide new insight into the mechanisms involved in *Salmonella* invasion of host cells.

## 1 INTRODUCTION

*Salmonella enterica* serovar Typhimurium (*S*. Typhimurium) is an intracellular bacterial pathogen and a major cause of foodborne gastroenteritis in humans (Mead et al., 1999). *Salmonella* uses a needle‐like apparatus known as a type three secretion system to translocate virulence proteins (effectors) into host cells (Kubori et al., 1998) that drive host cytoskeletal rearrangements and signalling pathways in order to promote bacterial invasion into nonphagocytic cells (Finlay, Ruschkowski, & Dedhar, 1991). Actin is essential for this process, since *S.* Typhimurium's ability to invade host cells is completely blocked by inhibitors that disrupt actin polymerisation such as cytochalasin D (Finlay et al., 1991). Many previous studies have focused on the regulation of actin dynamics during *S*. Typhimurium invasion (LaRock, Chaudhary, & Miller, 2015; Ly & Casanova, 2007; Truong, Copeland, & Brumell, 2014). However, the mechanisms of actin structural and temporal regulation during invasion are not completely understood.

Septins are small Guanosine triphosphate (GTP) binding proteins that associate with membranes and interact with microfilaments and microtubules to regulate their functions (Fung, Dai, & Trimble, 2014). Mammals have 13 septin encoding genes (Pan, Malmberg, & Momany, 2007; Sirajuddin et al., 2007), which can be separated into subgroups based on sequence homology. Septins have the ability to oligomerise into filaments and higher order structures (Kinoshita, Field, Coughlin, Straight, & Mitchison, 2002) in order to perform cellular functions such as microtubule stabilisation (Nagata et al., 2003), actin bundling (Schmidt & Nichols, 2004), as well as the formation of membrane diffusion barriers (Barral, Mermall, Mooseker, & Snyder, 2000), and molecular scaffolds (Joo, Surka, & Trimble, 2007). Septins have previously been implicated in processes such as cytokinesis in budding yeast (Kim, Haarer, & Pringle, 1991) and mammalian cells (Surka, Tsang, & Trimble, 2002), microbial infection (Phan et al., 2013; Torraca & Mostowy, 2016), phagocytosis (Huang et al., 2008) and endo‐lysosomal sorting (Song, Russo, & Krauss, 2016). Septins play a role in the internalisation of bacterial pathogens including Listeria monocytogenes (Pizarro‐Cerda et al., 2002), and here, we demonstrate a role for septins in the *S*. Typhimurium invasion process. We show that septins promote bacterial uptake via a pathway involving ROCK2 and Formin Homology 2 Domain Containing 1 (FHOD1).

## 2 RESULTS

### 2.1 Septins are recruited to *S.* Typhimurium invasion sites

**Figure 1:**
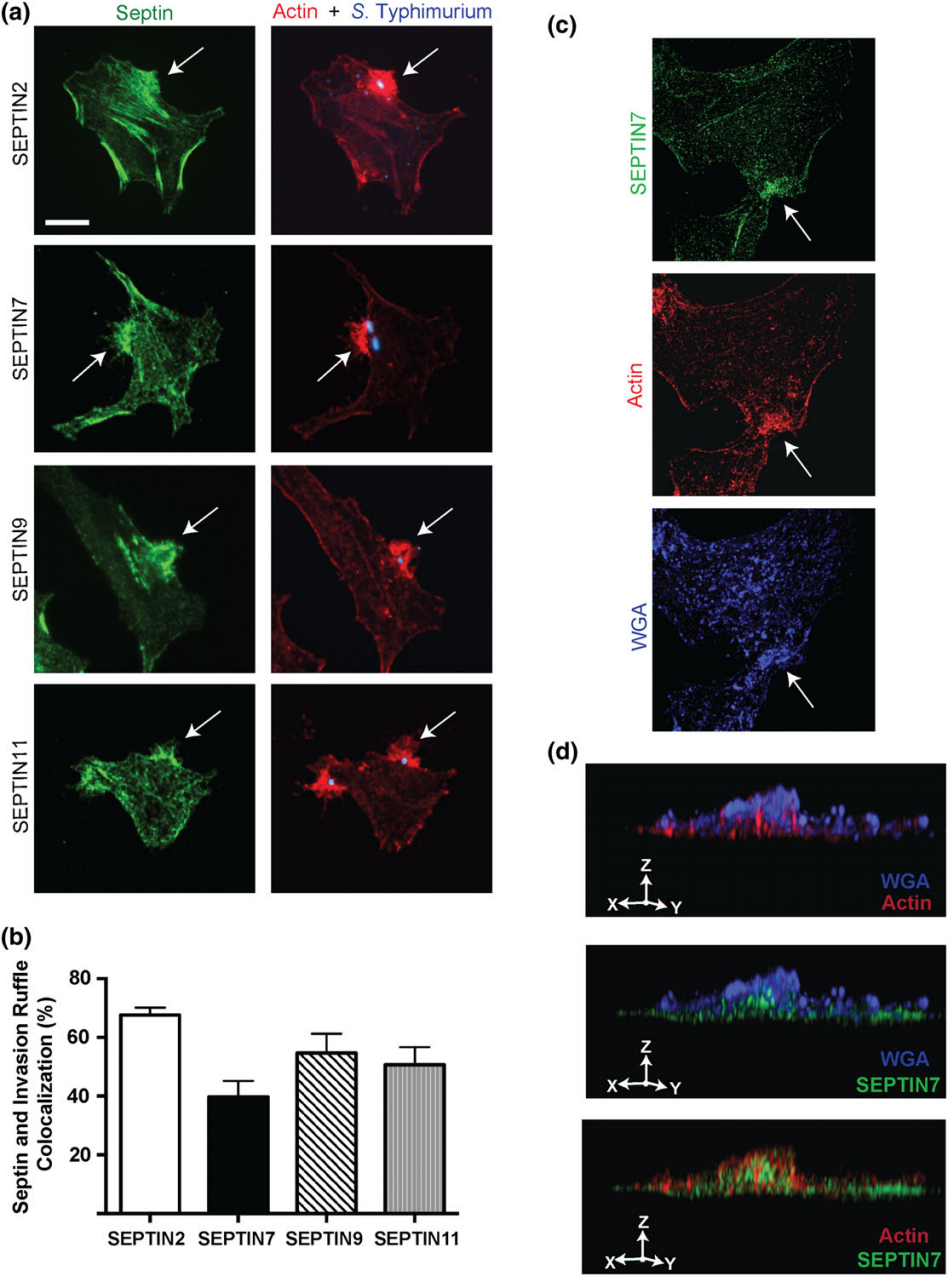
Septins are recruited to *Salmonella enterica* serovar Typhimurium invasion sites. Septin recruitment to the invasion ruffle was assessed in HeLa cells. (a) HeLa cells were infected with *S*. Typhimurium and fixed 10‐min postinvasion. Cells were then immunostained for endogenous SEPTIN2, SEPTIN7, SEPTIN9, and SEPTIN11 (green); F‐actin (red); and *S*. Typhimurium (blue). Scale bar, 11 μm. Images were taken using a spinning‐disk confocal microscope and invasion site is indicated by an arrow. (b) Quantification of septin recruitment to the invasion ruffle was done in 100 infected cells in three independent experiments. (c) Septin domain localisation to the invasion ruffle was assessed in HeLa cells using structured illumination microscopy. HeLa cells were infected with *S*. Typhimurium and fixed 10‐min postinvasion. Cells were then immunostained for endogenous SEPTIN7 (green), F‐actin (red), and the membrane marker wheat germ agglutinin WGA (blue). (d) 3D reconstruction of the membrane protrusion during invasion was created using image Z stacks. Area examined using 3D reconstruction is indicated by arrow

We examined the localisation of septins during *S*. Typhimurium invasion using affinity‐purified antibodies to SEPTIN2, SEPTIN7, SEPTIN9, and SEPTIN11. In control cells, endogenous septin proteins localised to cortical actin and stress fibres (Estey, Ciano‐Oliveira, Froese, Bejide, & Trimble, 2010; Joo et al., 2007; Surka et al., 2002). When cells were infected with *S*. Typhimurium for 10 min, we observed that septins were recruited to actin‐enriched invasion sites (see arrows in Figure 1a). Septin recruitment was observed in approximately 40%–60% of invasion sites (Figure 1b), possibly reflecting the dynamic nature of actin polymerisation at this site.

For high‐resolution visualisation of actin and septins during *S*. Typhimurium invasion, HeLa cells were infected with *S*. Typhimurium for 10 min, and invasion ruffles were examined by structured illumination microscopy (SIM; Figure 1c). For these experiments the plasma membrane was labelled with wheat germ agglutinin (WGA), which binds to cell surface N‐acetylglucosamine and N‐acetylneuraminic acid residues (Wright, 1984). Following 3D reconstruction, actin was found to occupy filamentous structures projecting dorsally from the cell surface in association with WGA+ plasma membrane (Figure 1d). Endogenous SEPTIN7 associated with these actin filaments in adjacent microdomains. These findings are consistent with prior studies indicating a role for septins in serving as a scaffold for F‐actin filaments.

### 2.2 Septins are required for efficient *S*. Typhimurium invasion of host cells

To determine whether the close association of septins with actin within the *S*. Typhimurium invasion ruffles affects bacterial internalisation, HeLa cells were treated with siRNA targeting *SEPTIN2*, *SEPTIN7*, or *SEPTIN9*. Depletion of septins was confirmed by Western blot analysis (Figure S1a,b). Cells were subsequently infected with *S*. Typhimurium for 30 min. In these experiments, we used a longer infection time to enrich for infected cells and to easily discern internalised bacteria. Immunostaining before permeabilisation was used to differentiate between intracellular and extracellular bacteria, as previously described by Smith et al. (2007).

**Figure 2:**
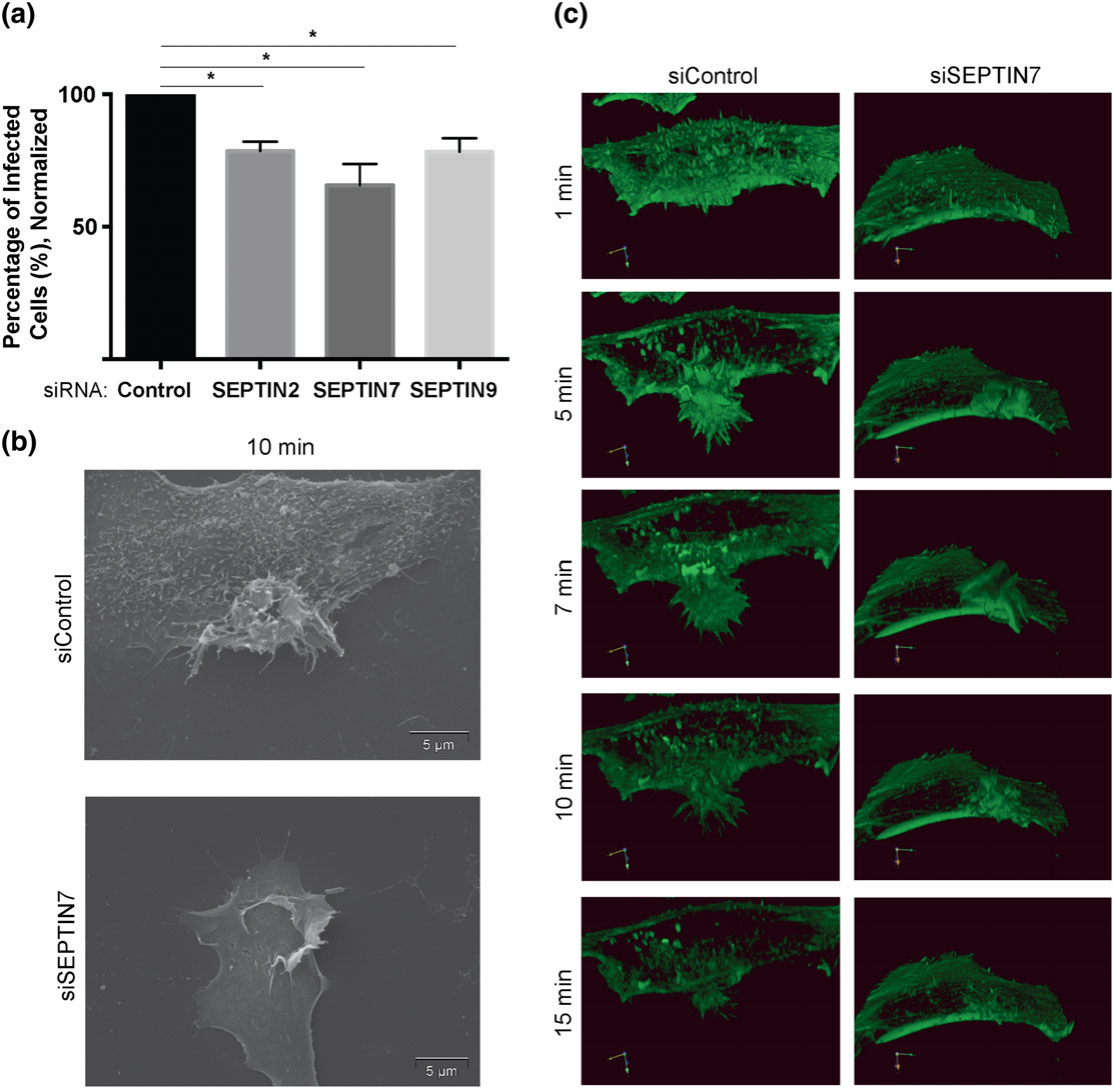
Septins are required for *Salmonella enterica* serovar Typhimurium invasion and affect invasion site morphology. (a) HeLa cells were transfected with the indicated siRNA for 48 hr. Post‐siRNA transfection, cells were infected with *S*. Typhimurium for 30 min. Differential antibody staining was used to identify intracellular and extracellular bacteria. 100 cells were analysed for bacterial infection. Data is normalised to cells treated with control siRNA. “*” denotes *p* value < 0.05. (b) Scanning electron microscopy of *S*. Typhimurium invasion sites upon septin knockdown. HeLa cells were transfected with the indicated siRNA and subsequently infected with *S*. Typhimurium for 10 min. Cells were then fixed with gluteraldehyde, and scanning electron microscopy images were taken at 8000x *g*. Scale bar, 5 μm. (c) Live cell imaging of *S*. Typhimurium invasion sites upon septin knockdown. HeLa cells were transfected with indicated siRNA. 24‐hr post‐siRNA knockdown, cells were transfected with LifeAct‐mRFP (shown in green) and then infected 24 hr later with wild ‐type *S*. Typhimurium. Invasion was recorded in live cells using spinning‐disk confocal microscopy. Times shown (in minutes) are relative to the initiation of the invasion process. Using Volocity analysis system, 3D reconstruction was produced to examine invasion ruffle formation under control and septin siRNA treated cells

Knockdown of SEPTIN2, SEPTIN7, and SEPTIN9 individually, caused a significant decrease in *S*. Typhimurium invasion relative to control siRNA‐treated cells (Figure 2a). An invasion defect was also observed when treating HeLa and Henle 407 cells with *SEPTIN7* siRNA pools (Figure S2a,b). Each siRNA pool contained two independent siRNAs targeting *SEPTIN7* and knockdown efficiency was confirmed (Figure S2c–f). Together, these results demonstrate a role for septins during the initial stages of *S*. Typhimurium infection.

SEPTIN7 is the stabilising septin within its hetero‐oligomeric complex (Fung et al., 2014). *SEPTIN7* siRNA treatment not only reduces levels of SEPTIN7 in the cell, but it also causes the destabilisation of other septin isoforms, unlike *SEPTIN2* and *SEPTIN9* siRNA (Figure S1b). For this reason, we employed *SEPTIN7* siRNA as a tool for subsequent studies of septin function during infection.

Since septin‐depleted cells have a significant bacterial internalisation defect, we examined the effect of septin knockdown on the morphology of invasion ruffles. Scanning electron microscopy (SEM) was used to obtain high‐resolution images of *S.* Typhimurium invasion sites (Figure 2b). HeLa cells were treated with the indicated siRNA and infected with *S.* Typhimurium for 10 min. Control siRNA‐treated cells displayed large membrane protrusions including finger‐like projections, known as filopodia, at the leading edge of the invasion ruffles. The cell surface also appeared rough with small filopodia‐like structures, consistent with previous studies (Truong et al., 2013). In contrast, knockdown of SEPTIN7 resulted in invasion ruffles with few filopodia, and the cell surface appeared smooth. Knockdown of SEPTIN7 also led to the formation of large sheet‐like protrusions (lamellipodia) that extended across the cell surface.

We also used live cell imaging to visualise the impact of SEPTIN7 knockdown on the morphology of invasion sites in real time. Cells were treated with *SEPTIN7* siRNA and transfected with LifeAct‐mRFP to visualise F‐actin. Cells were then infected with *S*. Typhimurium, and the invasion process was visualised using spinning‐disk confocal microscopy. Invasion sites of control siRNA treated cells displayed dynamic formation of filopodia and lamellipodia that are associated with bacterial uptake (Figure 2c, Supplemental Movie 1). The invasion sites in SEPTIN7‐depleted cells had minimal filopodia formation and produced extensive lamellipodia (Figure 2c; Supplemental Movie 2). These invasion site protrusions did not coalesce into typical membrane protrusions as observed in control cells. Rather, the lamellipodial protrusions dissipated in an outward fashion from the invasion initiation site. The invasion protrusions in SEPTIN7 knockdown cells resolved at a faster rate in comparison with the control cells: invasion sites were found to take approximately 25–30 min to resolve in control siRNA‐treated cells compared with 15–20 min in *SEPTIN7* siRNA‐treated cells. The shorter resolving time of invasion sites in SEPTIN7 knockdown cells suggests that septins could be involved in providing structural stability to the invasion ruffle and/or promoting the activity of actin nucleating factors.

### 2.3 Septins promote ROCK2 recruitment to *S*. Typhimurium invasion sites

Previous studies have demonstrated that septins function as a molecular platform to bring myosin and myosin kinases in close proximity for maximal myosin activation during contractile processes (Joo et al., 2007). RhoA‐associated kinases (ROCKs) are well‐studied activators of myosin II‐mediated contractility (Wilkinson, Paterson, & Marshall, 2005). ROCKs can directly phosphorylate myosin light chain II (MLC2) on Ser19 and Thr18 to promote myosin II activation, therefore promoting actomyosin contractility. This pathway is important for the RhoA‐myosin II‐dependent but Arp2/3 independent pathway, of *S*. Typhimurium invasion (Hanisch, Kolm, Wozniczka, Bumann, & Rottner, 2011). Interestingly, ROCK2 has been specifically implicated in the *S*. Typhimurium invasion process through its role in phosphorylating and activating FHOD1, an actin nucleator that promotes filopodia production (Truong et al., 2013). Thus, we examined the effect of SEPTIN7 knockdown on localisation of ROCK2 to the invasion ruffle during *S*. Typhimurium invasion.

**Figure 3:**
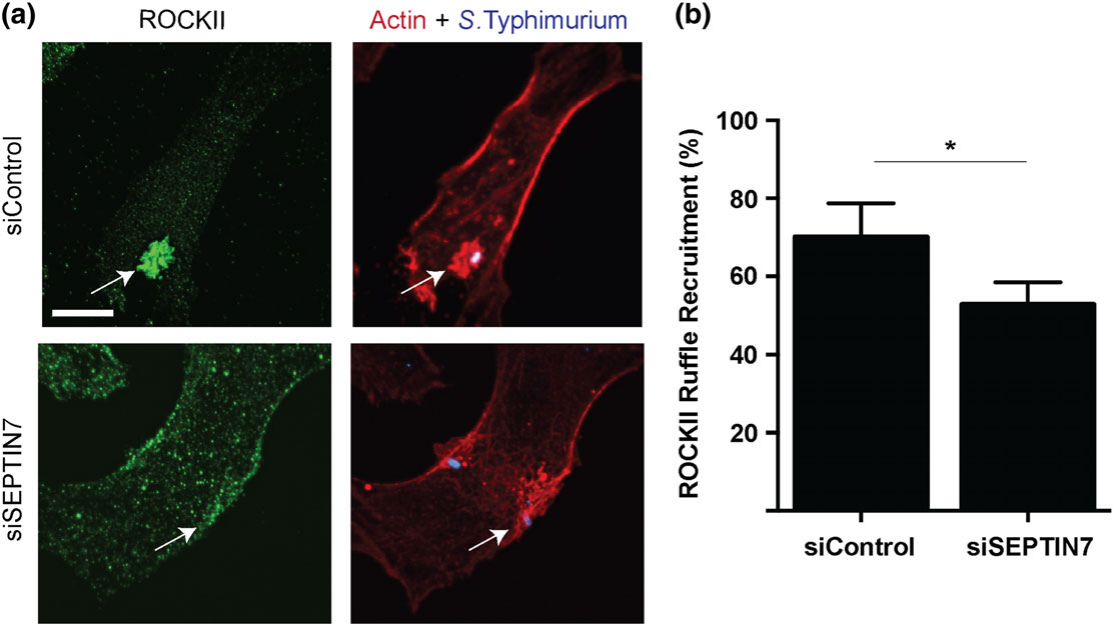
Septins promote ROCK2 recruitment to *Salmonella enterica* serovar Typhimurium invasion sites (a) HeLa cells were transfected with indicated siRNA. 48‐hr posttransfection, cells were infected with *S*. Typhimurium and fixed 10‐min postinvasion. Cells were then immunostained for endogenous ROCK2 (green), F‐actin (red), and bacteria (blue). Scale bar, 11 μm. Images were taken using spinning‐disk confocal microscopy and invasion site is indicated by an arrow. (b) Quantification of ROCK2 recruitment to the invasion ruffles under control, and SEPTIN7 knockdown conditions was done in 100 infected cells in three independent experiments. “*” denotes *p* value < 0.05

HeLa cells were treated with *SEPTIN7* siRNA 48 hr prior to infection. Subsequently, cells were infected with *S*. Typhimurium for 10 min. Endogenous ROCK2 colocalised with actin‐enriched invasion sites in control siRNA treated cells (Figure 3a, upper panels), an observation consistent with previous findings (Truong et al., 2013). Following SEPTIN7 knockdown, we observed a decrease in the frequency of ROCK2 recruitment to the invasion ruffle (Figure 3a,b). Therefore, septins contribute to ROCK2 recruitment to *S*. Typhimurium invasion sites. Impairment of ROCK2 recruitment to invasion sites could affect ROCK2‐dependent activation of myosin II and FHOD1, which are ROCK2 substrates known to play roles in *S*. Typhimurium invasion (Hanisch et al., 2011; Truong et al., 2013)**.** Thus, we examined the effect of SEPTIN7 knockdown on activation of ROCK2 substrates during *S*. Typhimurium invasion.

#### 2.3.1 Myosin II recruitment and activation during *S.* Typhimurium invasion does not require septins

Nonmuscle myosin II is a major contributor to cellular organisation and regulation. Myosins are able to crosslink and bundle actin filaments to promote contraction forces capable of deforming cell membranes in processes such as cell migration and phagocytosis (Newell‐Litwa, Horwitz, & Lamers, 2015). Prior studies by Stradal and colleagues characterised a RhoA/Myosin II‐dependent pathway of actin rearrangement during *S*. Typhimurium invasion (Hanisch et al., 2011). Myosin II was demonstrated to localize to *S.* Typhimurium invasion sites where it contributes to internalization of the bacteria. (Hanisch et al., 2011). Phosphorylation is required for Myosin II activity during contractile actions and it is known that myosin II phosphorylation occurs near sites where septin filaments are associated with actin stress fibres (Joo et al., 2007). Since septins can bind to septin‐associated Rho guanine nucleotide exchange factor (SA‐Rho‐GEF) and myosin, a signalling cascade of SA‐Rho‐GEF‐RhoA‐ROCK‐myosin II, which is essential for complete myosin II activation and thus myosin‐actin interaction, could be enabled by septin scaffolding (Nagata & Inagaki, 2005). Thus, it is possible that septins contribute to the localisation or activation of myosin II during *S*. Typhimurium invasion.

**Figure 4:**
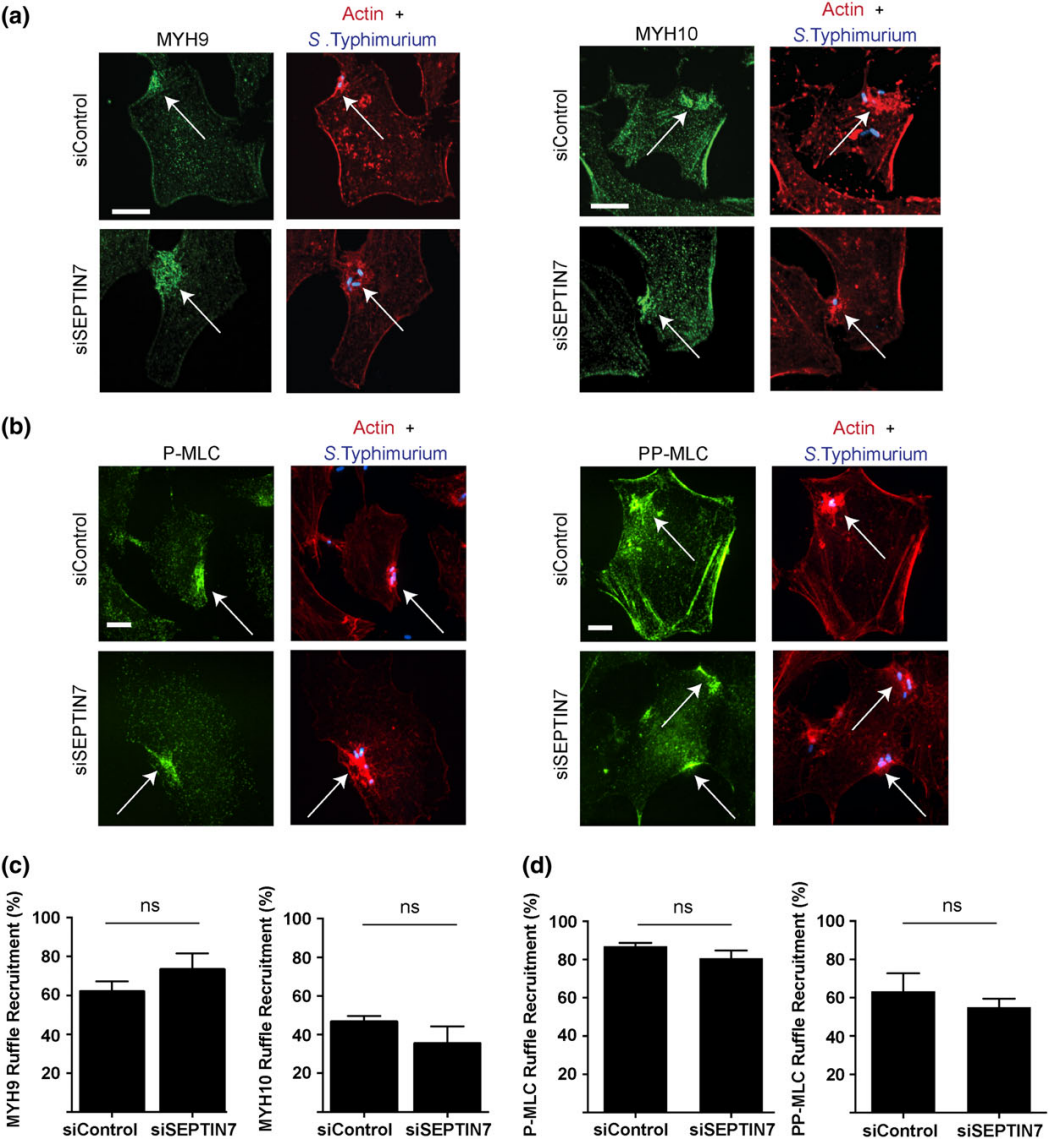
Myosin II recruitment and activation during *Salmonella enterica* serovar Typhimurium invasion does not require septins (a) HeLa cells were transfected with indicated siRNA. 48‐hr posttransfection, cells were infected with *S*. Typhimurium and fixed 10‐min postinvasion. Cells were then immunostained for endogenous MYH9 or MYH10 (green), F‐actin (red), and bacteria (blue). Scale bar, 11 μm. (b) HeLa cells were transfected with indicated siRNA. 48‐hr posttransfection, cells were infected with *S*. Typhimurium and fixed 10‐min postinvasion. Cells were then immunostained for endogenous P‐MLC or PP‐MLC (green), F‐actin (red), and bacteria (blue). Scale bar, 7 μm. Images were taken using spinning‐disk confocal microscopy and invasion site is indicated by an arrow. (c) Quantification of MYH9 and MYH10 recruitment to the invasion ruffle in control and SEPTIN7 knockdown conditions was counted in 100 infected cells in three independent experiments. (e) Quantification of P‐MLC and PP‐MLC recruitment to the invasion ruffle in control and SEPTIN7 knockdown conditions was counted in at least 100 infected cells in three independent experiments

To examine whether myosin II requires septins for localisation to the invasion ruffle, we tested the recruitment of the myosin II heavy chain isoforms, MYH9 and MYH10, under control and *SEPTIN7* siRNA knockdown conditions. At 48 hr post transfection, HeLa cells were infected with *S*. Typhimurium for 10 mins. We observed that endogenous MYH9 and MYH10 colocalised with the F‐actin enriched sites under control and SEPTIN7 knockdown conditions (Figure 4a,c). These results indicate that MYH9 and MYH10 do not require septins for localisation to *S*. Typhimurium invasion sites.

Myosin II can only promote contraction of actomyosin filament bundles when its myosin light chain (MLC) subunit is in the phosphorylated state (MLC; Newell‐Litwa et al., 2015). Since SEPTIN7 knockdown cells showed a decrease in ROCK2 recruitment to the invasion ruffle, we examined the recruitment of active MLC to the *S*. Typhimurium invasion site. HeLa cells were transfected with control or *SEPTIN7* siRNA and infected with *S*. Typhimurium for 10 mins. Following fixation, phospho‐specific antibodies to P‐MLC (Ser19) or PP‐MLC (Thr18/Ser19) were used to detect phosphorylated MLC. Both P‐MLC and PP‐MLC were enriched in *S*. Typhimurium invasion ruffles under both control and SEPTIN7 knockdown conditions (Figure 4b,d). Together, our findings indicate that septins are not required for myosin II recruitment or activation during *S*. Typhimurium invasion.

#### 2.3.2 Septins promote FHOD1 phosphorylation during *S*. Typhimurium invasion

**Figure 5:**
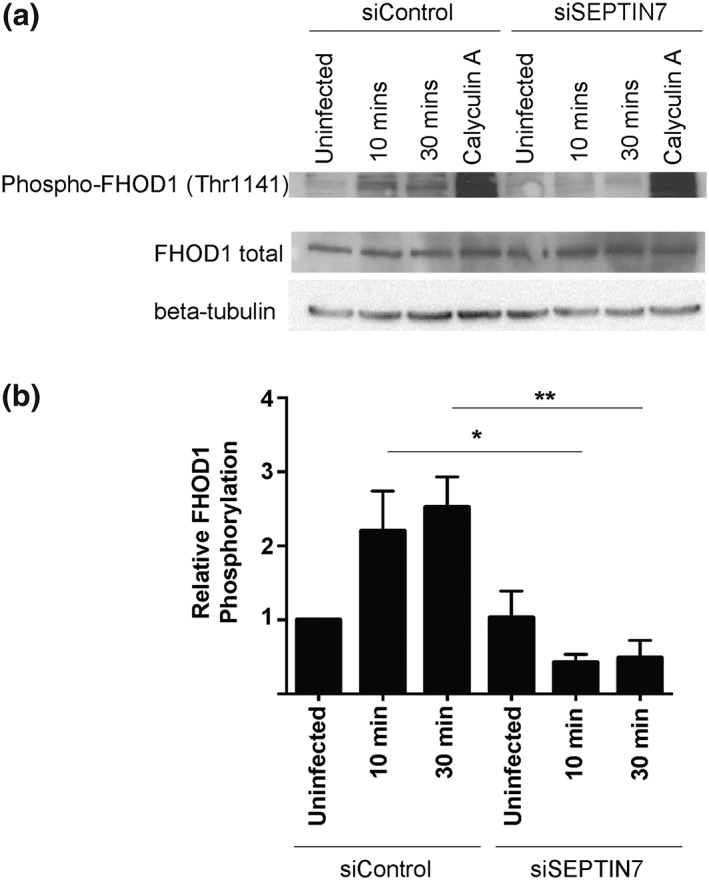
Septins promote FHOD1 phosphorylation during *Salmonella enterica* serovar Typhimurium invasion. (a) HeLa cells were transfected with the indicated siRNA. Cells were then infected with *S*. Typhimurium and lysed at the indicated time points. Whole cell lysates were probed with a phospho‐FHOD1 (Thr1141) antibody and total FHOD1 antibody. Treatment of cells with 50 nM Calyculin A (Serine/Threonine phosphatase inhibitor) was used as a positive control. (b) Densitometry was performed for three independent experiments. Levels of FHOD1 phosphorylation are normalised to total FHOD1 and represented relative to the uninfected control siRNA treated cells. “*” denotes *p* value < 0.05, “**” denotes *p* value < 0.01

FHOD1, a formin family member and host cell actin nucleating protein, has been shown to drive filopodia formation during the initial stage of *S*. Typhimurium invasion (Truong et al., 2013). Moreover, ROCK2 specifically mediates FHOD1 phosphorylation and activation during *S*. Typhimurium invasion (Truong et al., 2013). Since SEPTIN7 knockdown decreased recruitment of ROCK2 to *S*. Typhimurium invasion sites and we observed a striking loss of filopodia in invasion ruffles of SEPTIN7 knockdown cells, we tested whether septins act as a molecular scaffold for FHOD1 recruitment and activation as well. To test this, we examined the FHOD1 phosphorylation state following *SEPTIN7* siRNA treatment. HeLa cells were treated with control or *SEPTIN7* siRNA and were infected 48‐hr posttransfection with *S*. Typhimurium and lysed at indicated time points postinvasion. The phosphatase inhibitor Calyculin A was used as a positive phosphorylation control. Whole cell lysates were probed using phospho‐FHOD1 (Thr1141) and total FHOD1 antibodies. An increase in FHOD1 phosphorylation was observed in control siRNA treated cells at 10 and 30 min postinfection (Figure 5a,b), consistent with prior observations (Truong et al., 2013). In SEPTIN7‐depleted cells, the increase in FHOD1 phosphorylation at 10 min and 30 min postinfection was not observed. Total FHOD1 levels did not change following siRNA treatment. Taken together, our data suggests that septins promote FHOD1 phosphorylation during *S*. Typhimurium infection, likely by promoting the recruitment of its upstream activating kinase ROCK2 at the *S*. Typhimurium invasion site.

## 3 DISCUSSION

It is known that septins are important during uptake of both inert particles (Huang et al., 2008) and live bacterial pathogens into mammalian cells (Mostowy et al., 2009; Mostowy et al., 2011). However, the mechanisms by which septins promote these internalisation events have been unclear. Here, we examined the role of septins in *S*. Typhimurium invasion of nonphagocytic HeLa and Henle 407 cells. Our data reveals septins to be a significant component of the host cytoskeleton in the internalisation of these bacteria.

Prior studies have indicated rapid actin polymerisation at *S*. Typhimurium invasion sites is an essential event for bacterial uptake (Finlay et al., 1991). Live cell imaging has revealed that the early steps of invasion include rapid extension of F‐actin rich filopodia, followed by generation of lamellipodial structures and contraction of these cell surface protrusive structures (Hanisch et al., 2011; Truong et al., 2013). Subsequently, the plasma membrane is invaginated, and bacteria are internalised into cells in *Salmonella*‐containing vacuoles (Finlay et al., 1991; Terebiznik et al., 2002). In our study, we observed rapid enrichment of septins at *S*. Typhimurium invasion sites and their association with F‐actin rich microdomains. Knockdown of SEPTIN7 was used as a tool to disrupt the septin cytoskeleton, since it is essential for stability of other septins (Tooley et al., 2009). Under these conditions, we observed dramatic alterations to invasion sites, including a loss of filopodia and disorganisation of the lamellipodial protrusions. Our data are consistent with septins serving as a scaffold for the organisation of polymerised actin. It is interesting to note that although SEPTIN7 knockdown disrupts the septin cytoskeleton, we observed that SEPTIN2 and 9 could still be recruited to *S*. Typhimurium invasion sites in SEPTIN7 knockdown cells (Figure S3). This is likely due to their ability to bind phospholipids such as phosphatidylinositol 4,5‐bisphosphate (PtdIns (4,5)2) (Zhang et al., 1999), which is temporally enriched in *S*. Typhimurium invasion ruffles.

Our data also suggests that septin molecular platforms promote signal transduction events at *S*. Typhimurium invasion sites. We find that ROCK2 recruitment to invasion sites and activation of its downstream target FHOD1 are impaired in SEPTIN7 knockdown cells. Although septin depleted cells were shown previously to be impaired in generating PP‐MLC mediated by ROCK2 (Joo et al., 2007) we did not see an inhibition of myosin activation in SEPTIN7 depleted cells during invasion. This suggests that kinases other than ROCK2 may contribute to myosin II activation during *S*. Typhimurium invasion. In this regard, *S*. Typhimurium has been shown to induce elevation of intracellular calcium during invasion (Ruschkowski, Rosenshine, & Finlay, 1992), which may mediate myosin II activation through calcium‐activated kinases. Also, the *S*. Typhimurium lipid phosphatase known as SopB has been shown to induce PP‐MLC activation when transfected in HeLa cells (Wasylnka et al., 2008). Data from Wasylnka et al. (2008), has suggested that SopB activates myosin II in order to control positioning of the *Salmonella* containing vacuole postinvasion. SopB is also an important bacterial effector in *S*. Typhimurium invasion, and it is possible that activation of PP‐MLC at the *S*. Typhimurium invasion site of SEPTIN7 depleted cells can be explained by a pathway involving SopB‐mediated myosin II activation.

In conclusion, our studies of the *S*. Typhimurium invasion process have shown that septins play a role in bacterial entry by affecting invasion ruffle morphology and signalling through the host actin nucleator FHOD1. Future studies are required to determine the specific contributions of individual septin family members during *S*. Typhimurium invasion. Our findings support the idea that septins function as molecular scaffolds, by contributing to the localisation and downstream signalling of factors involved in regulating cytoskeletal dynamics. This data shows that intracellular bacterial pathogens can be useful tools for elucidating the molecular machinery involved in septin signalling. Future studies will bring additional insight into the intricate process of *Salmonella* invasion with the discovery of the molecular mechanisms that are essential for septin biological functions.

## 4 EXPERIMENTAL PROCEDURES

### 4.1 Cell culture, transfection and plasmids

HeLa and Henle 407 cells were obtained from American Type Culture Collection (ATCC) and maintained in growth medium (DMEM high glucose [HyClone] supplemented with 10% Fetal Bovine Serum (FBS) [Wisent]) in a 37 °C incubator with 5% CO_2_. HeLa cells were seeded at 5 × 10^4^ cells per well in 24‐well tissue culture plates containing 2.5 cm acid‐washed coverslips (except for cells destined for western blot analysis) or at 4.5 × 10^5^ cells per 6 cm dish 24 h before treatment.

Cells prepared for super resolution microscopy were cultured onto acid/alcohol washed high precision coverslips, 1.5‐mm thickness (Marienfed, 0117580). Calyculin A (EMD Millipore, 208851) was used to pre‐treat cells, where indicated.

For transfection of HeLa cells, Xtreme Gene 9 (Roche) transfection reagent was used as indicated by the manufacturer's protocol. Transfection of a LifeAct‐mRFP (Munsie et al., 2009) was used for live cell imaging.

### 4.2 RNA interference

HeLa and Henle 407 cells were seeded into 24‐well culture plates at 5 × 10^4^ cells per well and transfected 24 hr later using Lipofectamine (Invitrogen). The control siRNA was siCONTROL Non‐Targeting siRNA #2 (Dharmacon). *SEPTIN7*‐directed siRNA (5'‐UAU AUG CUG CAC UGA AUG GAA dT dT −3′) used in this work was obtained from Dharmacon. The siRNAs used in supplemental experiments (Sigma) were *SEPTIN7*‐directed siRNA 5' GAUAAUGAAAGAAAUCCAA dT dT −3' and 5'GACUGUACAGGUGGAACAA dT dT −3′ (siRNA Pool #1) and *SEPTIN7*‐directed siRNA 5' CAAUCUCCCAAAUCAAGUA dT dT −3′ and 5' CACUAUGAGAACUACAGAA dT dT −3′ (siRNA Pool #2). A concentration of 100 nM of total siRNA was used in each knockdown. Medium was changed 24 hr after transfection, and HeLa or Henle 407 cells were infected with *S*. Typhimurium 48 hr posttransfection.

### 4.3 Bacterial strains and infection

*S*. Typhimurium SL1344 was used in this study (Hoiseth & Stocker, 1981). Late‐log bacterial cultures were used for infecting HeLa and Henle 407 cells during experiments as outlined previously (Szeto, Namolovan, Osborne, Coombes, & Brumell, 2009). Briefly, WT bacteria were grown for 16 hr at 37 °C with shaking and then subcultured (1:33) in LB without antibiotics for 3 hr. Post subculture, bacteria were pelleted at 10,000 g for 2 min and re‐suspended in PBS with calcium and magnesium, pH 7.2. The inoculum was diluted and added to HeLa cells at 37°C for the indicated time points. For invasion experiments, bacteria were diluted to 1:50. For experiments probing for phosphorylated protein post‐septin knockdown, bacteria were diluted to 1:10. The cells were then washed at least 3 times with PBS with calcium and magnesium and fixed at time points indicated.

### 4.4 Live cell imaging

Cells were grown on 2.5‐cm coverslips, transfected 12–16 hr before invasion with LifeAct‐mRFP constructs and preincubated with RPMI‐1640 media (supplemented with L‐glutamine, HEPES, no bicarbonate; Wisent) with 10% FBS at 37 °C for 20 min. Cells were infected with WT SL1344 bacteria. In brief, 1 ml of late log bacterial suspension was extensively washed with PBS. The bacterial suspension was used for infection. Time‐lapse confocal z‐stacks of the cells were imaged using a Leica DMI 6000B inverted fluorescence microscope with a Hamamatsu ImagEMx2 camera (Quorum Technologies Inc., Guelph, Canada). Images were processed using Volocity 6 software (PerkinElmer).

### 4.5 Scanning electron microscopy

Cells were seeded in a 24‐well tissue culture plate at a density of 5 × 10^4^ cells per well and transfected with the indicated siRNA 24 hr later. Invasion was carried out 48 hr after transfection. Cells were infected with *S*. Typhimurium at the indicated time points. Samples were fixed in 2% glutaraldehyde in cacodylate buffer, rinsed in buffer and dehydrated in a graded ethanol series. The samples were critical point dried in a Bal‐tec CPD030 critical point dryer, mounted on aluminium stubs, gold coated in a Denton Desk II sputter coater and examined in an FEI XL30 SEM.

### 4.6 Antibodies

Western blotting and immunofluorescence staining of endogenous septins was performed with anti‐SEPTIN2 (Proteintech Group, 11397‐1), anti‐SEPTIN7 (Cedarlane, 13818‐1), anti‐SEPTIN9 and anti‐SEPTIN11 (Estey et al., 2010; Estey et al., 2013; Tsang et al., 2011). Salmonella O antisera (BD Difco) was used for immunostaining of *S*. Typhimurium (Cat No. 225341). Anti‐MYH9 (Cell signalling, 3403S) and anti‐MYH10 (Cell signalling, 3404S) were used for immunostaining myosin IIA and myosin IIB, respectively. Anti‐phospho‐myosin light chain 2 (Ser19; Cell signalling, #3671) and anti‐phospho‐myosin light chain 2 (Thr18/Ser19; Cell signalling, #3674) were used for immunofluorescence analysis of myosin phosphorylation. Anti‐phospho‐FHOD1 (Thr‐1141; ECM Bioscience, FP3481) and anti‐FHOD1 (ECM Bioscience, FM3521) were used to immunostain and probe for FHOD1 activity. Immunofluorescence staining of ROCK2 was completed with anti‐ROCK II (Upstate, 05‐841). Anti‐ß‐tubulin (Sigma Cat. No. T4026), and anti‐GAPDH (Millipore, Cat No. MAB374) were used to validate loading for Western blot analysis. All fluorescent secondary antibodies were AlexaFluor conjugates from Molecular Probes (Invitrogen).

### 4.7 Immunofluorescence microscopy

Cells were fixed with 1 or 2.5% paraformaldehyde in 1 x PBS for 10 min at 37 °C. Fixed cells were immunostained as previously described (Brumell, Rosenberger, Gotto, Marcus, & Finlay, 2001). Immunostaining before permeabilisation was used to differentiate between intracellular and extracellular bacteria (Smith et al., 2007). Coverslips were mounted onto glass slides using DakoCytomation fluorescence mounting medium and imaged using a Leica DMIRE2 inverted epifluorescence microscope or a Quorum spinning disk confocal microscope (Leica DMI6000B inverted fluorescence microscope, Hamamatsu ORCA Flash 4 sCMOS and colour camera) and processed using Volocity 6 software (Perkin Elmer).

For Structured Illumination Microscopy (SIM), high precision glass coverslips with 1.5‐mm thickness were mounted onto glass slides using Prolong Diamond Antifade Mountant and acquired on Zeiss Elyra PS1 equipped with an Axio Observer Z1 microscope, Andor iXon3 885 detectors and 60 × /1.4 NA plan‐Apochromat oil immersion objectives. Z‐stacks were collected and computationally deconvolved using Zeiss Zen 2012 with SIM licence.

### 4.8 Western blots

Cells were lysed in Lysis Buffer (1% Triton‐X 100, 50 mM Tris pH 7.4, 150 mM NaCl, and 1 mM EDTA) and Lysis buffer was supplemented with protease inhibitors (10 μg/ml aprotinin, 10 μg/ml leupeptin, 1 μM pepstatin A, 1 mM PMSF) and 1 mM Dithiothreitol (DTT). Sample buffer (60 mM Tris pH 6.8, 5% glycerol, 1% SDS, 2% β‐mercaptoethanol, 0.02% bromophenol blue) was added to the suspension, and samples boiled for 6 min. Samples were separated on 8% SDS‐PAGE gel for phospho‐FHOD1 blots and 12% for septin blots, then they were transferred to Polyvinylidene Fluoride (PVDF) membranes. Membranes were blocked in 3% BSA or 5% milk in TBS‐T overnight. Primary antibodies were incubated overnight at 4°C. Secondary antibodies used were conjugated to horseradish peroxidase (HRP) and were purchased from Sigma. Densitometry was performed on scanned immunoblot images using the ImageJ gel analysis tool (Abramoff, Magalhaes, & Ram, 2004).

### 4.9 Statistical analysis

Statistical analyses were conducted using GraphPad Prism v5.0. The mean +/− standard error of the mean is shown in figures, and *p* values were calculated using one sample *t* test or one‐way analysis of variance (ANOVA), where indicated. A *p* value of less than 0.05 was considered statistically significant and is denoted by *. *p* < 0.01 is denoted by ** and *p* < 0.001 is denoted by ***.

## CONFLICT OF INTEREST

The authors declare no conflict of interest.

## Supporting information

Supplemental Figure 1. **Knockdown of *SEPTIN2*, 7 and 9 expression with siRNA.** HeLa cells were transfected with the indicated siRNA. 48 h post siRNA transfection, cells were lysed and lysates were prepared as described in Methods and Experimental Procedures. (A) HeLa cells were transfected with *SEPTIN2* or *SEPTIN9* siRNA. Lysates were probed with antibody against endogenous SEPTIN2 and SEPTIN9. Equal loading was confirmed with an antibody against GAPDH. (B) HeLa cells were transfected with *SEPTIN7* siRNA. Lysates were probed with antibody against endogenous SEPTIN2, SEPTIN7, and SEPTIN9. Equal loading was confirmed with an antibody against GAPDH.

Supplemental Figure 2. **Knockdown of Septin expression with siRNA in HeLa and Henle 407 cells.** HeLa and Henle 407 cells were transfected with *SEPTIN7* siRNA pools. Each siRNA pool is made of two independent siRNA's targeting *SEPTIN7*. (A) 48 h post‐siRNA transfection, cells were infected with *S*. Typhimurium for 30 min. Differential antibody staining was used to identify intracellular and extracellular bacteria. 50 cells were analyzed for bacterial infection in at least 3 independent experiments. Data is normalized to cells treated with control siRNA. * denotes p‐value< 0.05. (B) 48 h post siRNA transfection, cells were lysed and lysates were prepared as described in Methods and Experimental Procedures. Lysates were probed with antibody against endogenous SEPTIN7 and equal loading was confirmed with an antibody against GAPDH. (C) Densitometry was performed for at least 3 independent experiments. SEPTIN7 levels were normalized to GAPDH and are represented relative to the control siRNA treated cells.

Supplemental Figure 3. **Recruitment of endogenous SEPTIN2 and 9 to *Salmonella* invasion ruffles in SEPTIN7 depleted cells**. HeLa cells were transfected with the indicated siRNA and 48 h post siRNA transfection septin recruitment to the invasion ruffle was assessed in HeLa cells. HeLa cells were infected with *S*. Typhimurium and fixed 10 min post‐invasion. Cells were then immunostained for endogenous SEPTIN2 and SEPTIN9 (green), F‐actin (red) and *S*. Typhimurium (blue). Scale bar, 6 μm. Images were taken using a spinning‐disk confocal microscope. Images were contrasted post‐imaging to adjust for lower signal intensity of SEPTIN2 and 9 in SEPTIN7 depleted cells

Supplemental Movie 1. **Dynamics of *S*. Typhimurium invasion sites in control siRNA treated cells.** HeLa cells were transfected with control siRNA for 24 h and then transfected with RFP‐LifeAct for 24 h. Cells were then infected with *S*. Typhimurium. Invasion was recorded in live cells using spinning‐disk confocal microscopy

Supplemental Movie 2. **Dynamics of *S*. Typhimurium invasion sites in *SEPT7* siRNA treated cells.** HeLa cells were transfected with *SEPTIN7* siRNA for 24 h and then transfected with RFP‐LifeAct for 24 h. Cells were then infected with *S*. Typhimurium. Invasion was recorded in live cells using spinning‐disk confocal microscopy
