# Supplementary figures and images for: Septin‐regulated actin dynamics promote Salmonella invasion of host cells
Source: Cell Microbiol. 2018 Jul 26;20(10):e12866. doi: 10.1111/cmi.12866 (PMC6175387; doi:10.1111/cmi.12866)

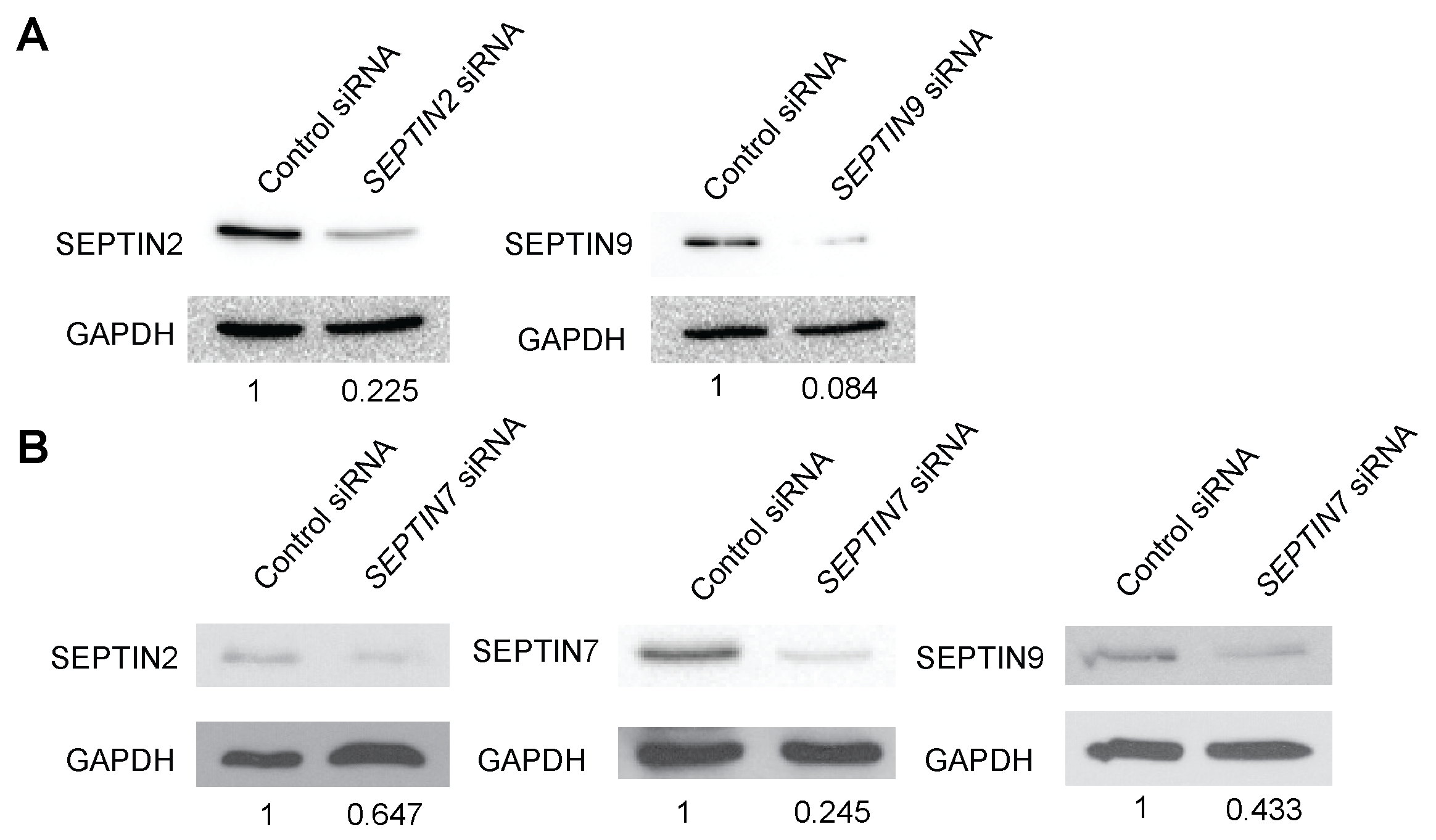

Supplement: Supplementary file 1 — Supplemental Figure 1. Knockdown of SEPTIN2, 7 and 9 expression with siRNA. HeLa cells were transfected with the indicated siRNA. 48 h post siRNA transfection, cells were lysed and lysates were prepared as described in Methods and Experimental Procedures. (A) HeLa cells were transfected with SEPTIN2 or SEPTIN9 siRNA. Lysates were probed with antibody against endogenous SEPTIN2 and SEPTIN9. Equal loading was confirmed with an antibody against GAPDH. (B) HeLa cells were transfected with SEPTIN7 siRNA. Lysates were probed with antibody against endogenous SEPTIN2, SEPTIN7, and SEPTIN9. Equal loading was confirmed with an antibody against GAPDH. [file CMI-20-na-s001.tif]

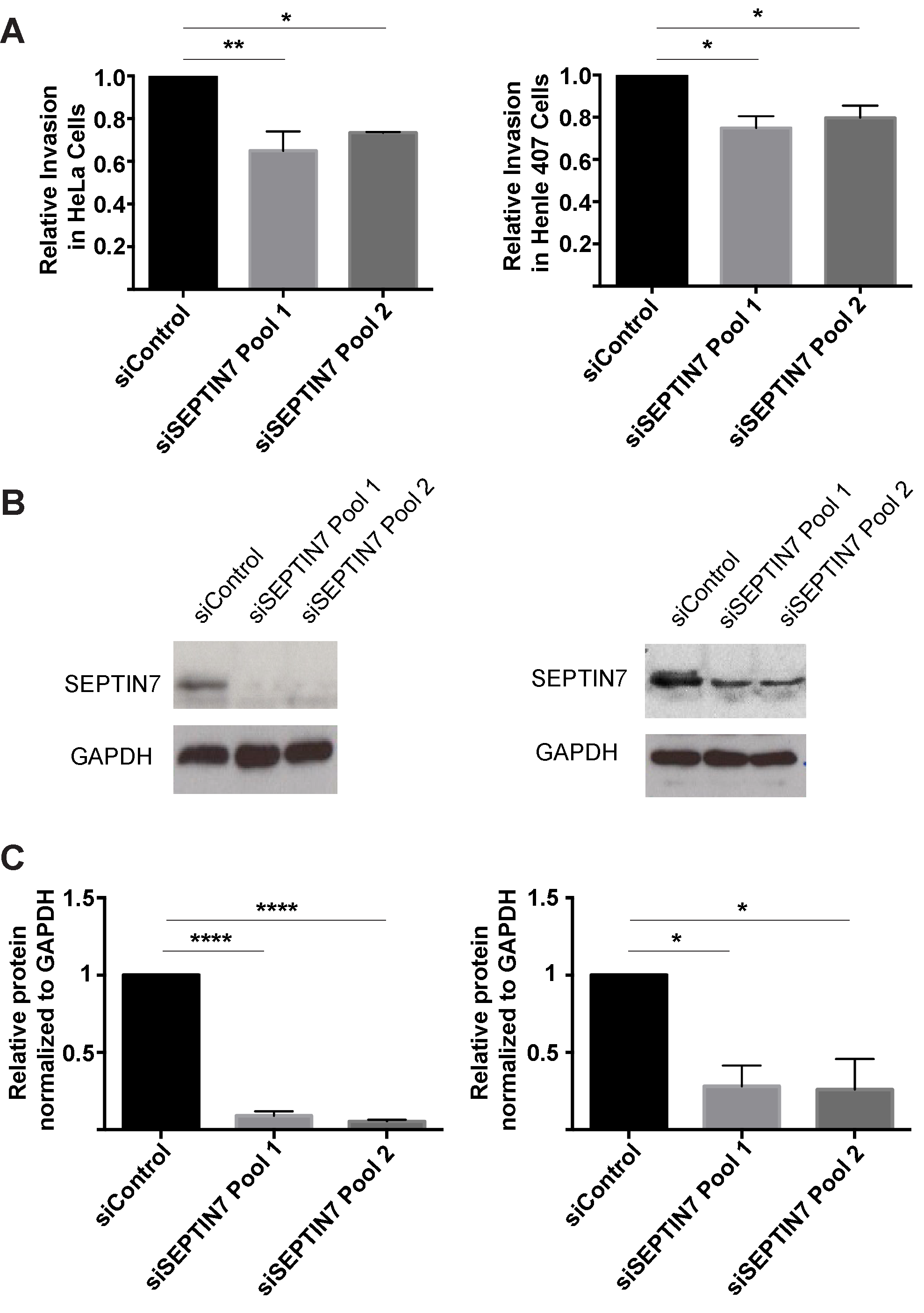

Supplement: Supplementary file 2 — Supplemental Figure 2. Knockdown of Septin expression with siRNA in HeLa and Henle 407 cells. HeLa and Henle 407 cells were transfected with SEPTIN7 siRNA pools. Each siRNA pool is made of two independent siRNA's targeting SEPTIN7. (A) 48 h post‐siRNA transfection, cells were infected with S. Typhimurium for 30 min. Differential antibody staining was used to identify intracellular and extracellular bacteria. 50 cells were analyzed for bacterial infection in at least 3 independent experiments. Data is normalized to cells treated with control siRNA. * denotes p‐value< 0.05. (B) 48 h post siRNA transfection, cells were lysed and lysates were prepared as described in Methods and Experimental Procedures. Lysates were probed with antibody against endogenous SEPTIN7 and equal loading was confirmed with an antibody against GAPDH. (C) Densitometry was performed for at least 3 independent experiments. SEPTIN7 levels were normalized to GAPDH and are represented relative to the control siRNA treated cells. [file CMI-20-na-s002.tif]

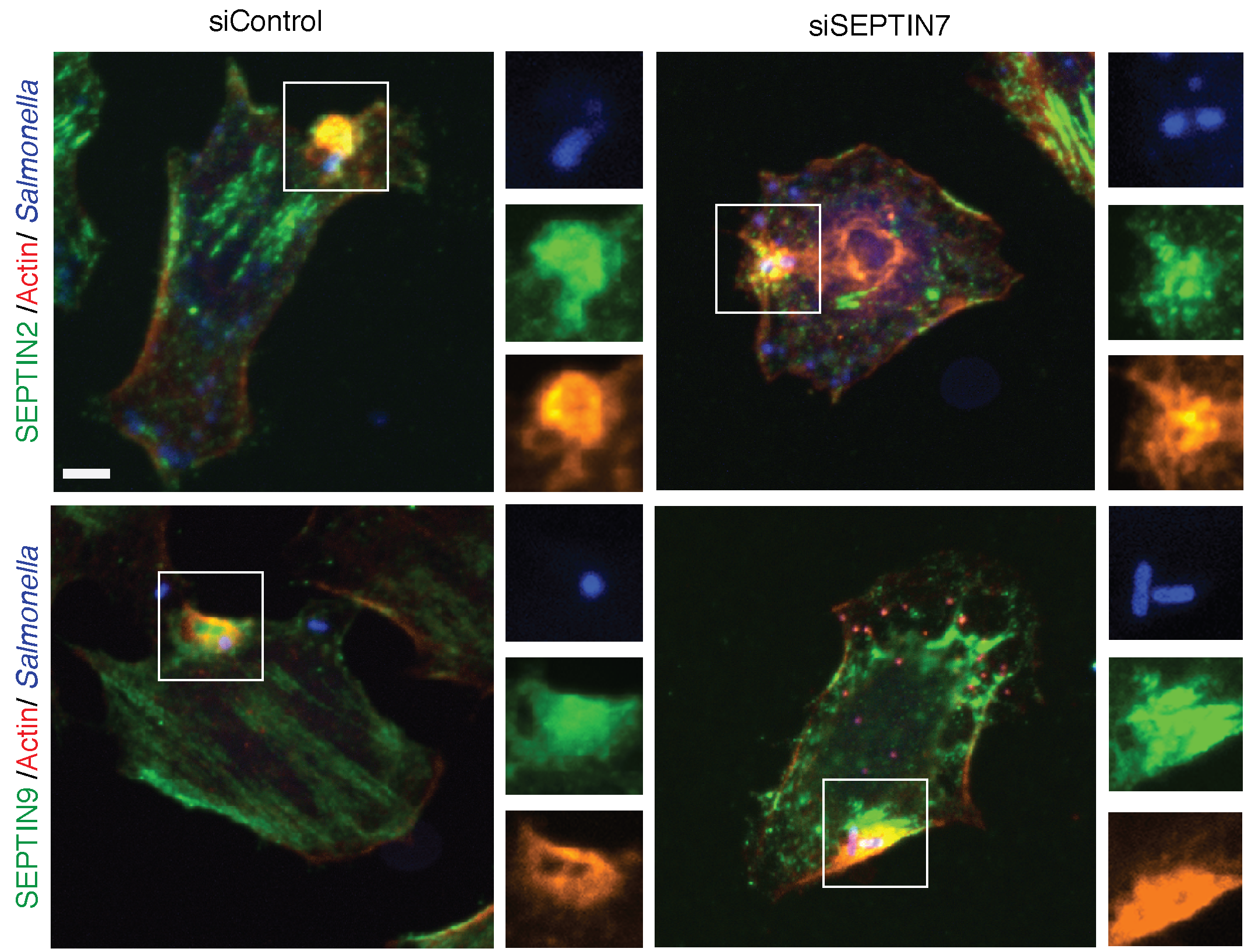

Supplement: Supplementary file 3 — Supplemental Figure 3. Recruitment of endogenous SEPTIN2 and 9 to Salmonella invasion ruffles in SEPTIN7 depleted cells. HeLa cells were transfected with the indicated siRNA and 48 h post siRNA transfection septin recruitment to the invasion ruffle was assessed in HeLa cells. HeLa cells were infected with S. Typhimurium and fixed 10 min post‐invasion. Cells were then immunostained for endogenous SEPTIN2 and SEPTIN9 (green), F‐actin (red) and S. Typhimurium (blue). Scale bar, 6 μm. Images were taken using a spinning‐disk confocal microscope. Images were contrasted post‐imaging to adjust for lower signal intensity of SEPTIN2 and 9 in SEPTIN7 depleted cells [file CMI-20-na-s003.tif]
